# Supplementary material for: Prognostic Impact of Tumor-Infiltrating Lymphocytes and Neutrophils on Survival of Patients with Upfront Resection of Pancreatic Cancer
Source: Cancers (Basel). 2019 Jan 3;11(1):39. doi: 10.3390/cancers11010039 (PMC6356339; doi:10.3390/cancers11010039)
Supplement: Supplementary file 1 [file cancers-11-00039-s001.pdf]

Supplementary Materials: Prognostic Impact of Tumor-Infiltrating Lymphocytes and Neutrophils on Survival of Patients with Upfront Resection of Pancreatic Cancer

Rainer C. Miksch, Markus B. Schoenberg, Maximilian Weniger, Florian Bösch, Steffen Ormanns, Barbara Mayer, Jens Werner, Alexandr V. Bazhin and Jan G. D'Haese

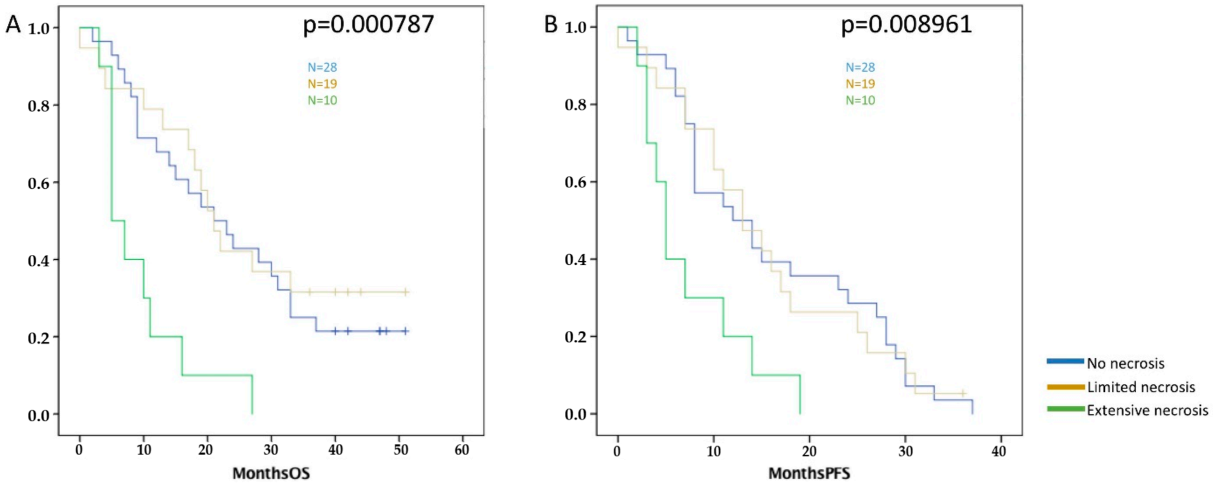

Figure S1. Extensive necrosis (grade 0–2) was correlated with impaired overall (**A**) and progression-free survival (**B**). Bigger tumor size was related with extensive grade of necrosis in the regression analysis (*p* = 0.000526).

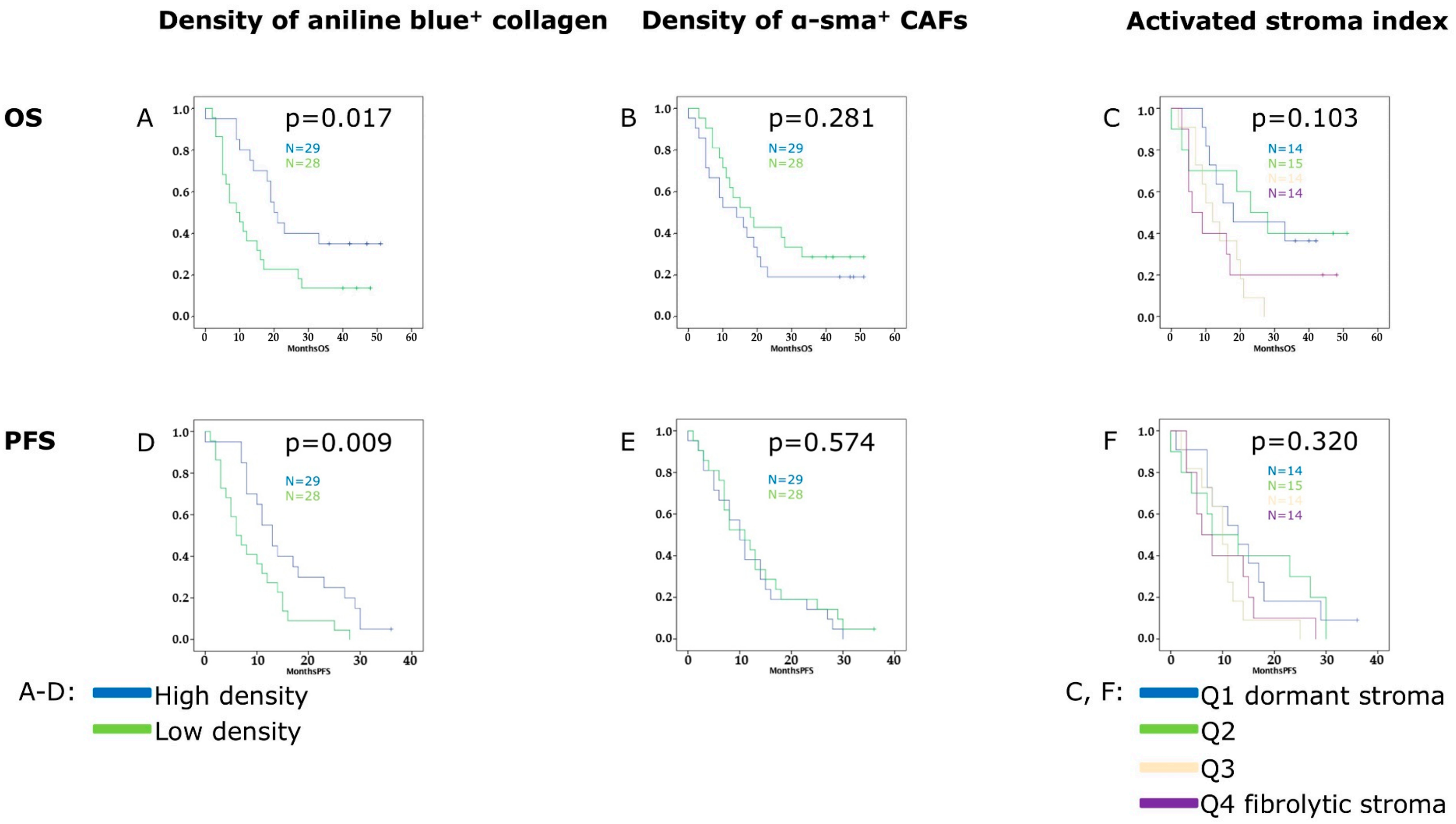

Figure S2. (**A**–**C**) Kaplan-Meier curves for overall survival (OS) and quality of the tumor stroma and for progression-free survival (PFS) and quality of the tumor stroma (**D**–**F**). A high density of aniline blue⁺ collagen areas was related significantly with OS ($p = 0.017$) and PFS ($p = 0.009$). A trend regarding OS can be described for high density of α-sma and cancer-associated fibroblasts (CAFs) and for a dormant stroma type in the activated stroma index.

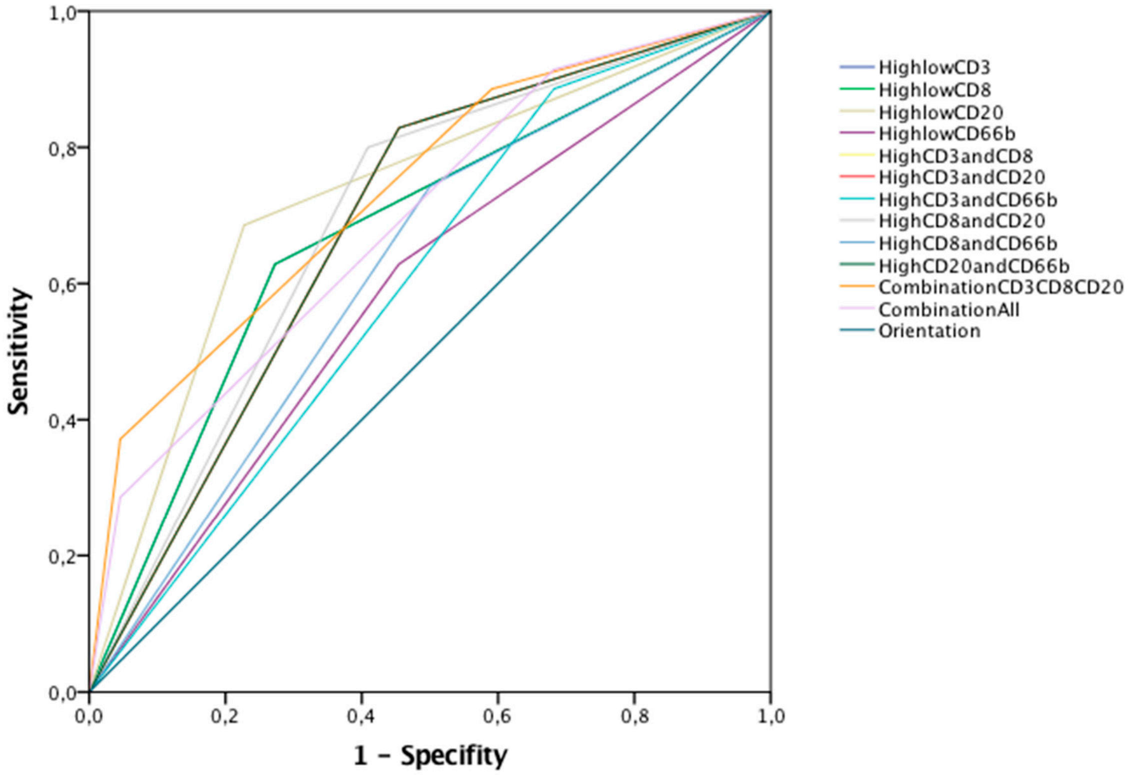

Figure S3. Receiver operating characteristic (ROC) curves for $CD3^+$, $CD8^+$, $CD20^+$, and $CD66b^+$ cells and combinations in relation to OS after 24 months. Here, the specificity and sensitivity of each cell type and combined immune cell groups are shown graphically. Evaluated tumor-infiltrating leukocytes help to predict survival two years after resection significantly (Table 3).

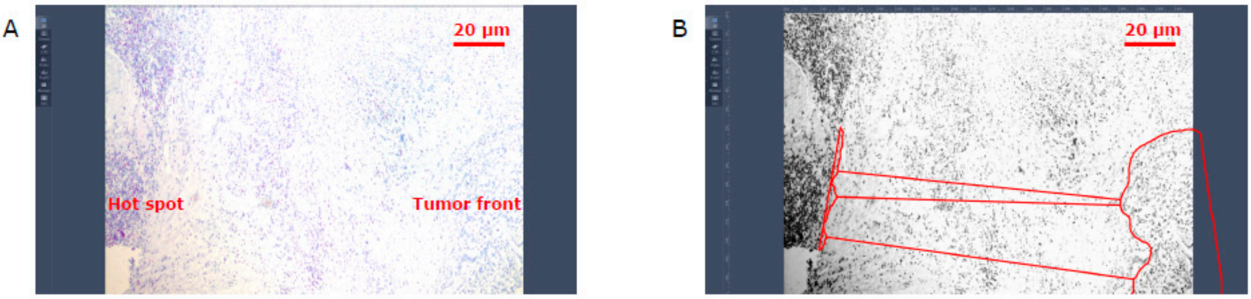

Figure S4. (**A**) On the left, a CD8+ TIL hot spot under a power field of 40× magnification with the microscope and on the right, the tumor front. (**B**) The distance between the hot spot and the tumor front was calculated using the ZEN 2 lite software (ZEN 2.0, Carl Zeiss, Jena, Germany), choosing 3 differences in this 2D-model and determining the mean value of the three.

© 2019 by the authors. Licensee MDPI, Basel, Switzerland. This article is an open access article distributed under the terms and conditions of the Creative Commons Attribution (CC BY) license (http://creativecommons.org/licenses/by/4.0/).
